# Supplementary material for: Do bromeliads affect the arboreal ant communities on orange trees in northwestern Costa Rica?
Source: PLoS One. 2022 Jul 6;17(7):e0271040. doi: 10.1371/journal.pone.0271040 (PMC9258856; doi:10.1371/journal.pone.0271040)
Supplement: Attachment — Submitted filename: Response to Reviewers.docx [file pone.0271040.s002.docx]

My main concern is that the authors do not explore the fact that there are species that establish intimate relationships with bromeliads (Who are these species?) and may have great potential in biological control (L13-15). See Anjos et al. (2021) Ants affect citrus pests and their natural

enemies in contrasting ways. Biological Control. <https://doi.org/10.1016/j.biocontrol.2021.104611>. These authors explore the potential of some ant species for biological control in citrus crops. I would like to see this issue further discussed in the "Discussion" section.

L28-38: We have summarized some of the important findings from the paper suggested by the reviewer in our introduction to help better contextualize our study : “Understanding these relationships can have important implications for the management of tropical agroecology operations, especially citrus orchards where ants are one of the most abundant insect groups [11]. A meta-analysis of the effects of ants on citrus pests and their natural enemies demonstrated the complexity of these relationships [11]. While ants are effective in controlling non-honeydew producing citrus pest abundance, their presence can also result in an increase in the abundance of honeydew-producing pests. That being said, the potential for ants to positively affect honeydew producers depends strongly on the identity of the ant species; *Lasius grandis –*an ecologically and behaviourally dominant ant species in Mediterranean citrus orchards–, for example, suppresses natural enemies of honeydew-producing pests by up to 145% relative to other less abundant ant species [11–13].

L361-367: We have added to the final paragraph of our discussion to further discuss the use of ants in the context of biological control, and to contextualize our findings through this lens: “In citrus orchards in particular, the potential of ants as biological control agents against crop pests is being actively explored, with results suggesting that ant presence is associated with increased abundance of honeydew-producing pests, but reduced abundance of pests that spend part of their lives in the soil. This reaffirms the importance of epiphytic plants like bromeliads in their role as a keystone resource in tropical systems [56], specifically as a correlate and potential determinant of ant community composition in the context of biological control.”

L20-21: See Rosumek et al. (2009). Ants on plants: a meta-analysis of the role of ants as plant biotic defenses. Oecologia, 160(3), 537-549.

L20-22: The following statement was added to better incorporate relevant information from the papers suggested by the reviewer (Rosumek et al. 2009) and the editor (Pacheco and Del-Claro, 2018): “These effects are particularly significant in tropical regions, where ant removal in plants resulted in a decrease in plant fitness of 59%, in part due to the role of ants in preventing herbivore damage [4,5].”

L21-22: Ants also establish important relationships with some caterpillar species (Lycaenidae). Cushman et al. (1994). Assessing benefits to both participants in a lycaenidâant association. Ecology, 75(4), 1031-1041.

L22-26: The proposed example of butterfly larva-tending behaviours was incorporated into the section identified by the reviewer.

L22: See Styrsky & Eubanks (2007). Ecological consequences of interactions between ants and honeydew-producing insects. Proceedings of the Royal Society B: Biological Sciences, 274(1607), 151-164.

L26-28: We thank the reviewer for suggesting this interesting and useful reference and have incorporated relevant details into our manuscript.

L23-24: and higher spread of disease by aphids.

L24-26: We have incorporated two relevant references that address the potential for aphids to act as vectors for plant diseases.

L42: See Camargo et al. (2012). Natural history of the Neotropical arboreal ant, Odontomachus hastatus: nest sites, foraging schedule, and diet. Journal of insect science, 12(1).

L50-52: We have incorporated this useful reference into our manuscript to highlight the potential of bromeliads to act as foraging sites in addition to nesting sites for ant species.

L69: I liked Figure 1 representing the predictions.

L94: How many bromeliads were there (on average) on the trees? What is the average size of the bromeliads removed? The size and shape of bromeliads determines some attributes of the tree ant communities.

L107: The following statement was included to address this comment: “On average, trees contained about 3 bromeliads each.”

L140-141: We added the follow statement to clarify the average diameter and longest leaf length of the dissected bromeliads to give a better idea of size and shape: “On average, bromeliads measured 6.6cm ± 0.2cm in diameter with an average longest leaf length of 52.1cm ± 1.5cm.”

L95: How was the removal of the bromeliads? With a climber? How can the authors be sure that all the large bromeliads (> 15 cm) were removed if the trees are relatively large and have a complex canopy?

L103-105: The following statement was included to address the reviewer’s concern: “Due to the shape of the tree canopies and the low height of the trees (2.5m to 4m), climbers were easily able to access bromeliads and visually ensure all large bromeliads were removed.”

L96: How many bromeliads were there (on average) on the trees? The average is about 3 bromeliads.

L107: The following statement was included to address this comment: “On average, trees contained about 3 bromeliads each.”

L97: Why this period? Is it enough time for the arthropod fauna to recover?

L109-110: The duration of the recovery period was chosen based on what has been done in similar published studies (see “Wetzel, W.C., Screen, R.M., Li, I., McKenzie, J., Phillips, K.A., Cruz, M., Zhang, W., Greene, A., Lee, E., Singh, N., Tran, C., Yang, L.H., 2016. Ecosystem engineering by a gall-forming wasp indirectly suppresses diversity and density of herbivores on oak trees. Ecology 97, 427438. <https://doi.org/10.1890/15-1347.1>”). This reference along with the following statement were added to the manuscript to address this comment: “The duration of recovery was chosen based on the timeframes used by other similar studies”.

L100-103: What is the estimated prevalence of large bromeliads (> 15cm) in orange groves?

L112-113: The following statement was added incorporated to address this comment: “Large bromeliads accounted for between one third and one half of the overall abundance of bromeliads on a given tree (longest leaf length ≥ 15cm.)”

L111: It is not a quadrant, but a cube (three dimensions), right? What was the structure of the cube?

L124-126: The original manuscript text was edited to clarify the dimensions of the sampling area, and the term “quadrat” was replaced by “polygon” throughout the text: “Within sites CP and ER, we set up four, three-dimensional sampling polygons (height: 50cm; width: 50cm; depth:100cm) directed towards the center each tree and sampled each polygon before (May 2017) and after (June-July 2017) the removal of bromeliads.”

L115: What distance?

L128-130: The distance was included in the following sentence: “…the first polygon was placed close to the tree’s largest bromeliad (at a distance of one quarter of the tree’s total circumference)”

L140-141: How did you look for nests or similar structures on the bromeliads? Did you locate alates, pupae and eggs in these structures?

L138-140: The following statement was included to provide a clearer description of how ant nests were identified: “The 174 bromeliads that were removed were dissected leaf by leaf, and the presence of ant nests was inferred based on the presence of workers, eggs, and larvae within the detritus within in a given bromeliad.”

L184: Table 1 does not show who these species are.

L214-215: This information is located in Table 2. Accordingly, we have added the following statement to the caption of Table 1: “Refer to Table 2 for a full list of ant species within each category.”

Figure 2. Does abundance have these low mean values?

The abundance values are low because they represent the mean abundances across 4 sampling polygons across all trees in a given treatment.

L191. I suggest an improvement in the formatting of the tables in the manuscript.

Table 2: Table formatting has been improved to clarify which species were considered bromeliad-associated, and which were not by splitting it into parts a) and b).

L227. The authors believe that Camponotus rectangularis may be a specialist bromeliad-nesting species? Do you have information on the natural history of this species? See Camargo, R. X., Oliveira, P. S., & Muscedere, M. (2012). Natural history of the Neotropical arboreal ant, Odontomachus hastatus: nest sites, foraging schedule, and diet. Journal of insect science, 12(1).

L325-329: We have added natural history information about this species as part of our discussion about the facultative use of bromeliads by some ant species: “Similarly, *Camponotus rectangularis* ­­­–a species we found was strongly associated with bromeliads– has previously been shown to nest in bromeliads [42–44]; however, it is also known to establish mutualistic relationships with the orchid *Myrmecophila tibicinis* [45] and nest in various other tree species [44,46].”

L240. Table 2. What is Ant BD, Ant BF, Ant Z and Ant AI?

L266-268: These ants were species that we were unable to identify and we have adjusted the table caption accordingly: “Morphospecies that we were not able to associate with a taxonomic name are indicated with ‘Ant’ followed by a letter code.”

Figure 4. Isn't it too much information to put the centroids on these graphs?

We feel that the centroids are important to include to ensure that the reader can more clearly see the overall patterns in species composition through the collapsing of many points into three areas.

I was curious to know how many ant species were sampled in total? And how many were considered bromeliad ants? I think this information should be in the text. Maybe I missed it, but I would also like to know who are the bromeliad antsâ¦ Perhaps it would be interesting to have a table (e.g., Supplementary Material) with the relative abundance of each ant species in the treatments considered in this manuscript.

L273-277: We have included the total number of species sampled as well as how many of these species were bromeliad-associated or not in the following section: “We found that the presence of bromeliads on orange trees was associated with modest effects on the composition of the total ant community (40 species) (Table 3). The bromeliad treatments differed in the subset of the ant community that was bromeliad-associated (14 species), but not the subset of ants that were never found nesting in bromeliads (26 species) (Table 3).”

L271-282: This paragraph is a bit confusing!

L296-306: We have rewritten this section as follows: “We found that orange trees with bromeliads had, compared to trees without bromeliads, moderately different species composition of the complete ant community (40 species) (Table 3). This pattern was repeated in the subset of the ant community that was bromeliad-associated (14 species), but not the subset of ants that were never found nesting in bromeliads (26 species) (Table 3). These results are in line with our prediction that bromeliad presence is associated with a change in ant species composition, and our prediction that bromeliad nesting ants account for this effect. By contrast, there was little support for our prediction that removing bromeliads would shift ant communities from resembling those on “with-with” trees (Before period) to more closely resembling without-without trees in the After period. Specifically, we found no significant sampling period x treatment interaction for any of the three ant categories (Table 3, Fig 4).”

L279: How far away from the forest were the selected trees? Was the edge effect not considered?

L91-92: The following statement was added to the “Study sites” section within the “Materials and methods” to provide some clarification as to why we did not directly consider edge effects: “Moreover, both sites were surrounded by forest such that the distance of a given tree to any edge varied between 50m and 100m.”

L286: Please, provide references.

L310: An appropriate reference was added.

L295-298: Isn't it a contradictory idea to link desiccation with an

increase in ant abundance in the dry season?

We meant that desiccation led to a redistribution of ants in the habitat, with ants congregating on water sources like bromeliads – and so being increasingly detected in our sampled trees with bromeliads. We have clarified this by using the term “local abundance” rather than “abundance” throughout this section.

L334: See Camargo et al. (2012). Natural history of the Neotropical arboreal ant, Odontomachus hastatus: nest sites, foraging schedule, and diet. Journal of insect science, 12(1).

L352-361: These results point to a potentially important role for bromeliads and their associated ants in tropical agroecosystems; however, the nature of their role may depend on the life history of the ant species, and on the biology of a given pest [11]. Ants have been shown to act as inducible defences against herbivory in mutualistic ant-plant systems such as the *Pseudomyrmex-Tachigali* system [5] and are recognized as having a significant role in the elimination of fungi and phytopathogens [51–53]. The presence of ants such as *Solenopsis* sp. A or *Camponotus rectangularis* that exhibit tending behaviours towards honeydew-producing may also have an indirect net positive effect on host plants by deterring non-honeydew producing herbivores [11]; however, these benefits have been found to decrease with leaf size in some systems [54] and be negated entirely by honeydew-producer herbivory in others [55].”
